# Supplementary material for: Mapping the anatomical and transcriptional landscape of early human fetal ovary development
Source: Sci Rep. 2025 May 6;15:15814. doi: 10.1038/s41598-025-96135-y (PMC12055976; doi:10.1038/s41598-025-96135-y)
Supplement: Supplementary file 1 — Supplementary Material 1 [file 41598_2025_96135_MOESM1_ESM.pdf]

Supplementary Materials

Mapping the anatomical and transcriptional landscape of early human fetal ovary development

Sinead M McGlacken-Byrne[1*], Ignacio del Valle[1], Theodoros Xenakis[1], Ian C Simcock[2,3,4], Jenifer P Suntharalingham[1], Federica Buonocore[1], Berta Crespo[5], Nadjeda Moreno[5], Danielle Liptrot[5], Paola Niola[6], Tony Brooks[6], Gerard S Conway[7], Mehul T Dattani[1], Owen J Arthurs[2,3,4], Nita Solanky[5], John C Achermann[1]

[1] Genetics and Genomic Medicine Research and Teaching Department, UCL Great Ormond Street Institute of Child Health, University College London, London WC1N 1EH, United Kingdom;

[2] Department of Clinical Radiology, Great Ormond Street Hospital for Children NHS Foundation Trust, London, WC1N 3JH, United Kingdom;

[3] Population, Policy and Practice Research and Teaching Department, UCL Great Ormond Street Institute of Child Health, University College London, London, WC1N 1EH, United Kingdom;

[4] NIHR Great Ormond Street Biomedical Research Centre, London, WC1N 1EH, United Kingdom;

[5] Developmental Biology and Cancer Research and Teaching Department, UCL Great Ormond Street Institute of Child Health, University College London, London WC1N 1EH, United Kingdom;

[6] UCL Genomics, Zayed Centre for Research, UCL Great Ormond Street Institute of Child Health, University College London, London WC1N 1DZ, United Kingdom;

[7] Institute for Women's Health, University College London, London, WC1E 6AU, United Kingdom.

Supplementary Files

Supplementary Movie 1: Micro-CT of human fetal ovary and oviduct (Fallopian tube) at 20wpc

Supplementary Materials: Supplementary figures 1-8

Supplementary Table 1: Width, length, and weight of the early human fetal ovary

Supplementary Table 2: Top 250 differentially expressed genes, ovary v testis.

Supplementary Table 3: Top 250 differentially expressed genes, ovary v control.

Supplementary Table 4: Top 250 differentially expressed genes, testis v ovary.

Supplementary Table 5: Top 250 differentially expressed genes, testis v control.

Supplementary Table 6: Top 250 differentially expressed genes, ovary v testis 15/16wpc.

Supplementary Table 7: Top 250 differentially expressed genes, ovary v testis 11/12wpc

Supplementary Table 8: Top 250 differentially expressed genes, ovary v testis 9/10wpc.

Supplementary Table 9: Top 250 differentially expressed genes, ovary v testis CS22/23.

Supplementary Table 10: Differentially expressed genes, ovary 15/16wpc v ovary CS22/23.

Supplementary Table 11: The 288 highly ovary specific genes.

Supplementary Table 12: Comparison of differentially expressed genes (DEGs) between the developing ovary and testis

Supplementary Table 13: Highly ovary-specific non-coding RNA genes.

Supplementary Table 14: Gene markers used for annotation of cell clusters.

Supplementary Table 15: MAGE cancer-testis genes in the developing ovary.

Supplementary Table 16: Differentially expressed transcription factors in the fetal gonad.

Supplementary Table 17: Differentially expressed nuclear receptor genes, ovary v control and ovary v testis.

Supplementary Table 18: Genes on the “PanelApp” POI panel designed by the 100,000 Genome Study, version 1.69, November 2024

Supplementary Methods 1:

R Packages used in scRNAseq analysis

tidyr_1.2.1	fansi_1.0.3	listenv_0.9.0	renv_0.16.0
abind_1.4-5	farver_2.1.1	lmtest_0.9-40	reshape2_1.4.4
AnnotationDbi_1.56.2	fastmap_1.1.0	locfit_1.5-9.7	reticulate_1.26
AnnotationHub_3.2.2	filelock_1.0.2	magrittr_2.0.3	rhdf5_2.38.1
assertthat_0.2.1	fitdistrplus_1.1-8	MASS_7.3-58.1	rhdf5filters_1.6.0
beachmat_2.10.0	future_1.30.0	Matrix_1.5-3	Rhdf5lib_1.16.0
Biobase_2.54.0	future.apply_1.10.0	MatrixGenerics_1.6.0	rlang_1.0.6
BiocFileCache_2.2.1	generics_0.1.3	matrixStats_0.63.0	ROCR_1.0-11
BiocGenerics_0.40.0	GenomeInfoDb_1.30.1	memoise_2.0.1	RSQLite_2.2.20
BiocManager_1.30.19	GenomeInfoDbData_1.2.7	mime_0.12	rstudioapi_0.14
BiocParallel_1.28.3	GenomicRanges_1.46.1	miniUI_0.1.1.1	Rtsne_0.16
BiocVersion_3.14.0	ggforce_0.4.1	munsell_0.5.0	S4Vectors_0.32.4
Biostrings_2.62.0	ggplot2_3.4.0	nlme_3.1-161	scales_1.2.1
bit_4.0.5	ggraph_2.1.0	parallel_4.1.0	scattermore_0.8
bit64_4.0.5	ggrepel_0.9.2	parallelly_1.33.0	sctransform_0.3.5
bitops_1.0-7	ggridges_0.5.4	patchwork_1.1.2	scuttle_1.4.0
blob_1.2.3	globals_0.16.2	pbapply_1.6-0	Seurat_4.3.0
cachem_1.0.6	glue_1.6.2	pillar_1.8.1	shiny_1.7.4
celldex_1.4.0	goftest_1.2-3	pkgconfig_2.0.3	SingleCellExperiment_1.16.0
cli_3.5.0	graphlayouts_0.8.4	plotly_4.10.1	sparseMatrixStats_1.6.0
cluster_2.1.4	grid_4.1.0	plyr_1.8.8	spatstat.data_3.0-0
clustree_0.5.0	gridExtra_2.3	png_0.1-8	spatstat.explore_3.0-5
codetools_0.2-18	gtable_0.3.1	polyclip_1.10-4	spatstat.geom_3.0-3
colorspace_2.0-3	HDF5Array_1.22.1	progressr_0.12.0	spatstat.random_3.0-1
compiler_4.1.0	htmltools_0.5.4	promises_1.2.0.1	spatstat.sparse_3.0-0
cowplot_1.1.1	htmlwidgets_1.6.0	purrr_1.0.0	spatstat.utils_3.0-1
crayon_1.5.2	httpuv_1.6.7	R.methodsS3_1.8.2	splines_4.1.0
curl_4.3.3	httr_1.4.4	R.oo_1.25.0	stringr_1.5.0
data.table_1.14.6	ica_1.0-3	R.utils_2.12.2	SummarizedExperiment_1.24.0
DBI_1.1.3	igraph_1.3.5	R6_2.5.1	survival_3.4-0
dbplyr_2.2.1	interactiveDisplayBase_1.32.0	RANN_2.6.1	tensor_1.5
DelayedArray_0.20.0	IRanges_2.28.0	rappdirs_0.3.3	tibble_3.1.8
DelayedMatrixStats_1.16.0	irlba_2.3.5.1	RColorBrewer_1.1-3	tidygraph_1.2.2
deldir_1.0-6	jsonlite_1.8.4	R.oo_1.25.0	tidyselect_1.2.0
digest_0.6.31	KEGGREST_1.34.0	R.utils_2.12.2	tools_4.1.0
DoubletFinder_2.0.3	KernSmooth_2.23-20	R6_2.5.1	tweenr_2.0.2
dplyr_1.0.10	labeling_0.4.2	RANN_2.6.1	utf8_1.2.2
dqrng_0.3.0	later_1.3.0	rappdirs_0.3.3	uwot_0.1.14
DropletUtils_1.14.2	lattice_0.20-45	RColorBrewer_1.1-3	vctrs_0.5.1
edgeR_3.36.0	lazyeval_0.2.2	Rcpp_1.0.9	viridis_0.6.2
ellipsis_0.3.2	leiden_0.4.3	RcppAnnoy_0.0.20	SoupX_1.6.2
euratObject_4.1.3	lifecycle_1.0.3	RCurl_1.98-1.9	ParamSweep_3.0
ExperimentHub_2.2.1	limma_3.50.3	remotes_2.4.2	SCTransform_0.3.5

Supplementary Methods 2:

Components for the buffers used for single-nuclei suspension:

Salty Ez10 Lysis Buffer

10 mM Tris-HCl pH 7.5 1M

146 mM NaCl 5M

1 mM $CaCl_2$ 1M

21 mM $MgCl_2$ 1M

0.03% Tween-20 (Sigma-Aldrich)

0.01% BSA (Miltenyi Biotec)

10% Ez Lysis Buffer (Sigma-Aldrich)

0.2-1 U/uL Protector RNAse Inhibitor (Roche)

1 mM DTT (ThermoFisher Scientific)

Wash and Resuspension Buffer 2 (WRB2)

10 mM Tris-HCl pH 7.5

10 mM NaCl

3 mM $MgCl_2$

1mM DTT (ThermoFisher Scientific)

1% BSA (Miltenyi Biotec)

0.2-1 U/uL Protector RNAse Inhibitor (Roche)

Supplementary Figure 1. Bulk RNA sequencing experimental design

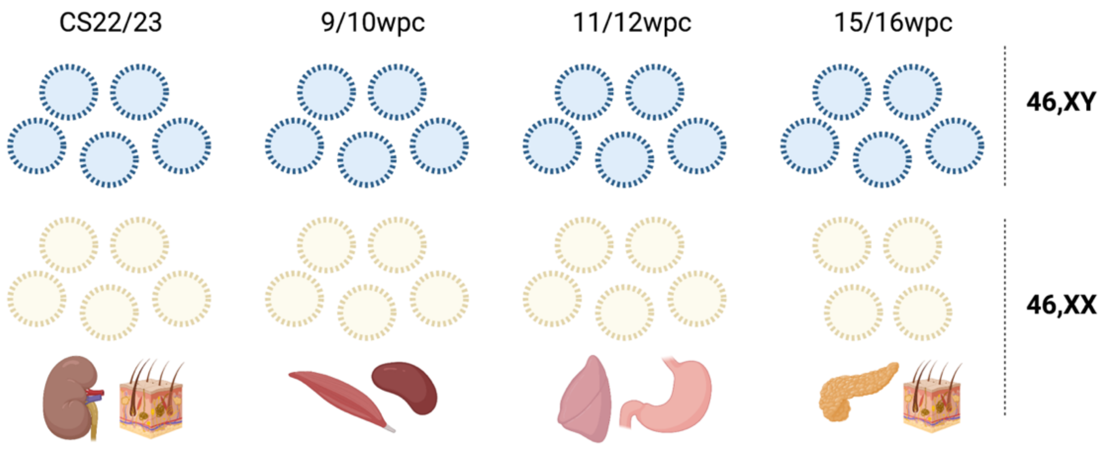

Testis (blue), ovary (yellow), and 46,XX control samples (kidney, skin, muscle, spleen, lung, stomach, pancreas) were collected for sequencing from each of four key developmental stages (CS22/23; 9/10wpc; 11/12wpc; 15/16wpc).

Supplementary Figure 2. Cluster analysis of experimental samples.

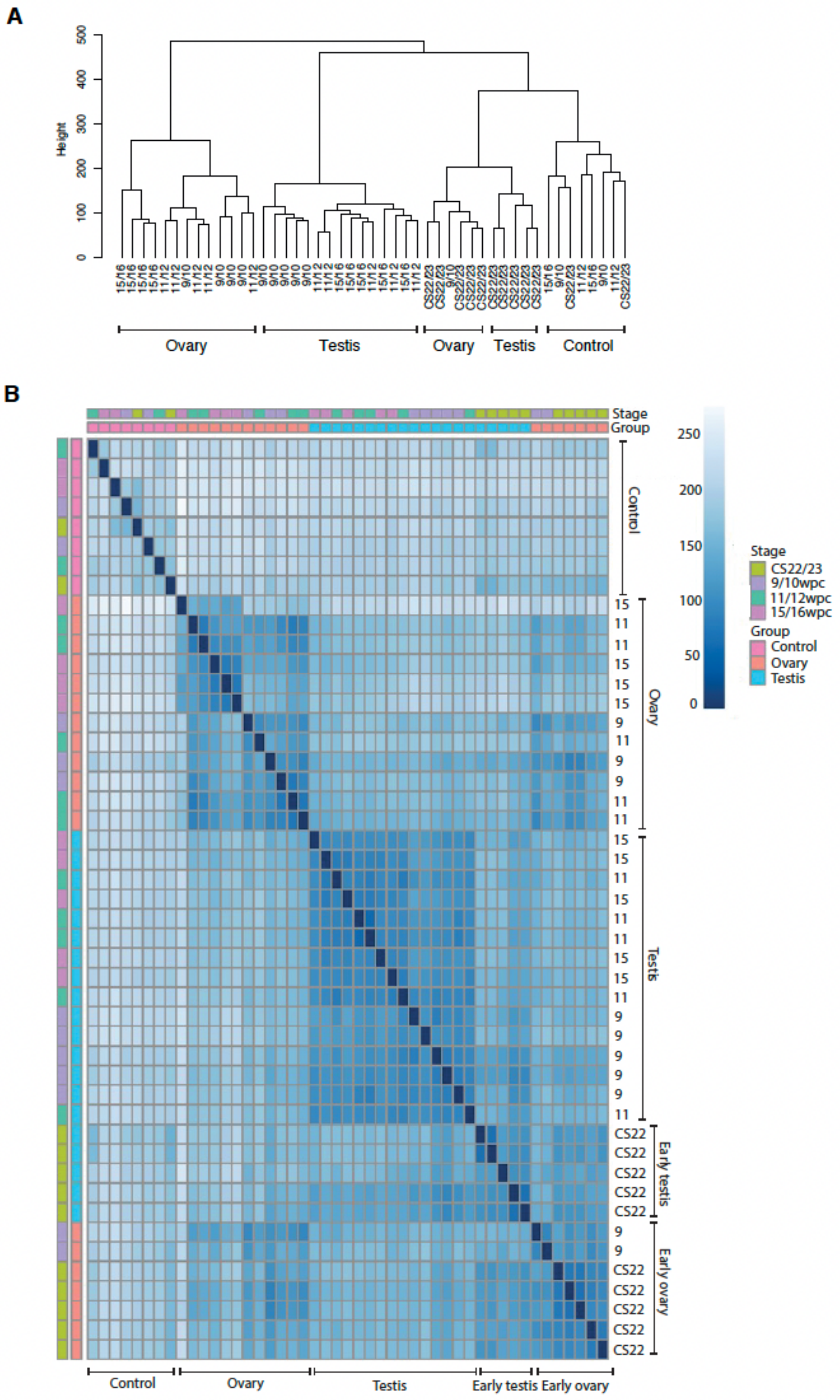

A) Cluster dendrogram of all 47 samples used in the study demonstrating clustering by developmental stage and tissue type. The hierarchical agglomeration clustering method (Ward's method, or ward.D2) was used for analysis**. B)** Correlation heatmap of gene expression across all 47 samples. Darker intensity and scores closer to 0 indicate higher correlation between samples.

Supplementary Figure 3. Non-coding transcripts in the developing fetal gonad.

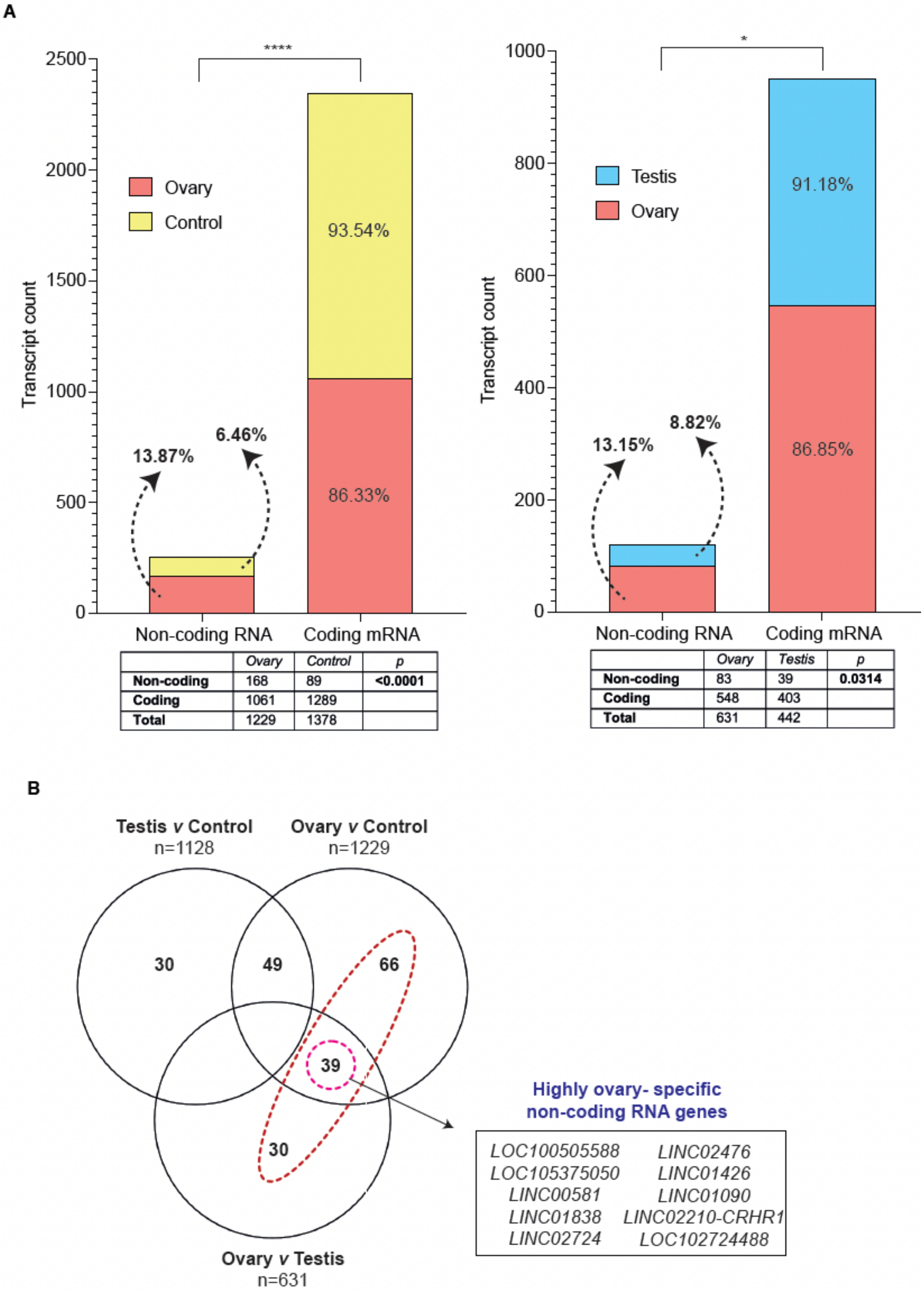

	Ovary	*Control*	*p*
**Non-coding**	168	89	**<0.0001**
**Coding**	1061	1289	
**Total**	1229	1378	

	Ovary	*Testis*	*p*
**Non-coding**	83	39	**0.0314**
**Coding**	548	403	
**Total**	631	442	

A) Total numbers of differentially expressed non-coding and coding transcripts between the ovary and control (left panel) and the ovary and testis (right panel) were examined globally ($log_2FC>2$; p.adj<0.05). Percentages of non-coding and coding transcripts compared to the total number of differentially expressed genes in the two analyses are indicated on both graphs. Differences in proportions of non-coding transcripts in the ovary compared to control or testis were examined using a Fisher's exact test (**p*<0.05; *****p*<0.0001). **B)** Absolute numbers of differentially expressed non-coding transcripts ($log_2FC>2$; p.adj<0.05) in the ovary compared to control; the testis compared to control; and the ovary compared to testis are shown. Ovary-specific (red) and highly ovary-specific transcripts (pink) are indicated. The top 10 differentially expressed highly ovary-specific non-coding RNA transcripts are indicated on the Venn diagram.

Supplementary Figure 4. Pathway enrichment analysis of genes differentially expressed in the ovary compared to control and to testis.

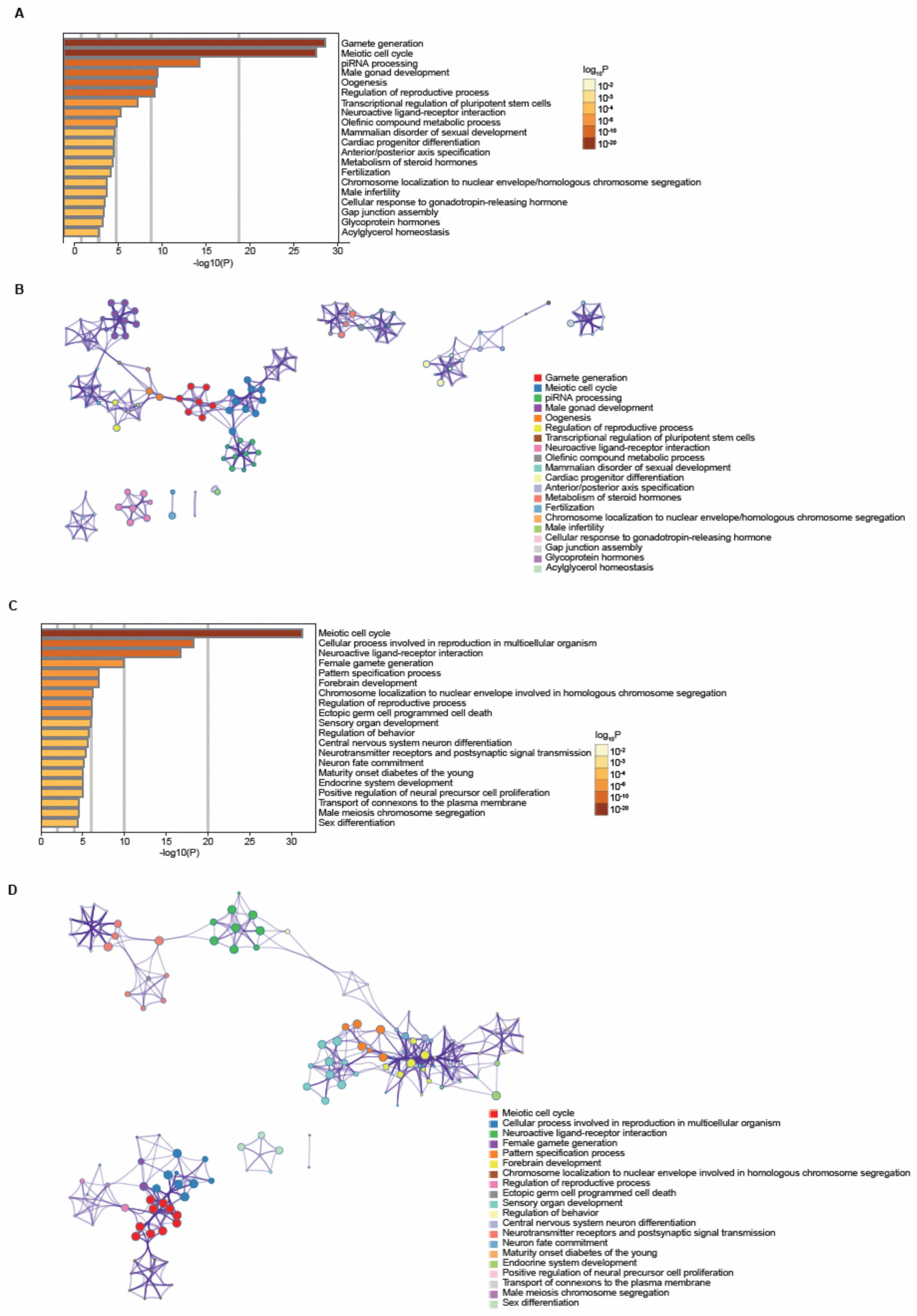

A) Bar graph of enriched gene ontology terms within the ovary *v* control analysis ($log_2FC>2$, p.adj<0.05; n=1229 total genes). Bars are coloured by $log_{10}p$ value with darker coloured bars indicating greater significance. **B)** Network analysis and visualisation of enriched gene ontology terms in the in the ovary *v* control analysis where each node represents an enriched term coloured by cluster ID (Metascape). Nodes with the same cluster ID cluster together. Terms with >0.3 similarity are connected by edges. **C)** Bar graph of enriched gene ontology terms within the ovary *v* testis analysis ($log_2FC>2$, p.adj<0.05; n=631 total genes). **D)** Network analysis and visualization of enriched gene ontology terms in the ovary *v* testis analysis.

Supplementary Figure 5: snRNAseq RNA sequencing quality control

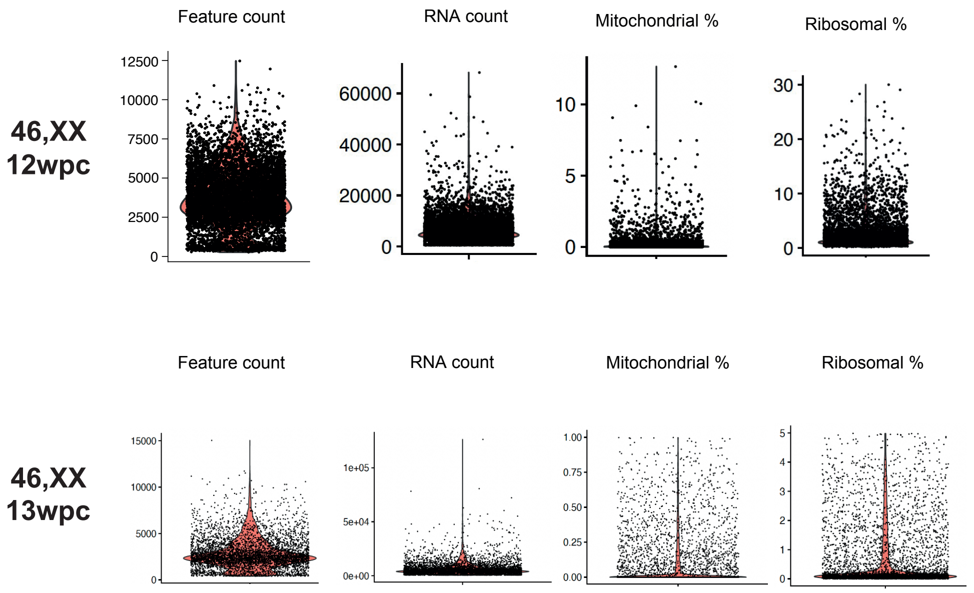

	13wpc 46,XX	*12wpc 46,XX*
Raw cells	5121	7033
<1% mitochondrial counts	4884	6745
<5% ribosomal counts	4732	6180
Unique feature counts >400	4705	6179
Doublets removed	4489	5802
**Final cell count**	**4489**	**5802**

Upper panel: For each of the four samples included in the snRNAseq analysis, the feature count, RNA count, percent mitochondrial genes and percent ribosomal genes are shown.

Lower panel: The starting number of raw cells is shown for each ovary sample. Cells were then removed from the analysis if they had >1% mitochondrial counts, >5% ribosomal counts, unique feature counts <400, or were identified as doublets. The final cell count for analysis is shown in bold.

Supplementary Figure 6: Validation of novel ovary-specific genes using the Garcia-Alonso *et al* single-cell gonadal atlas

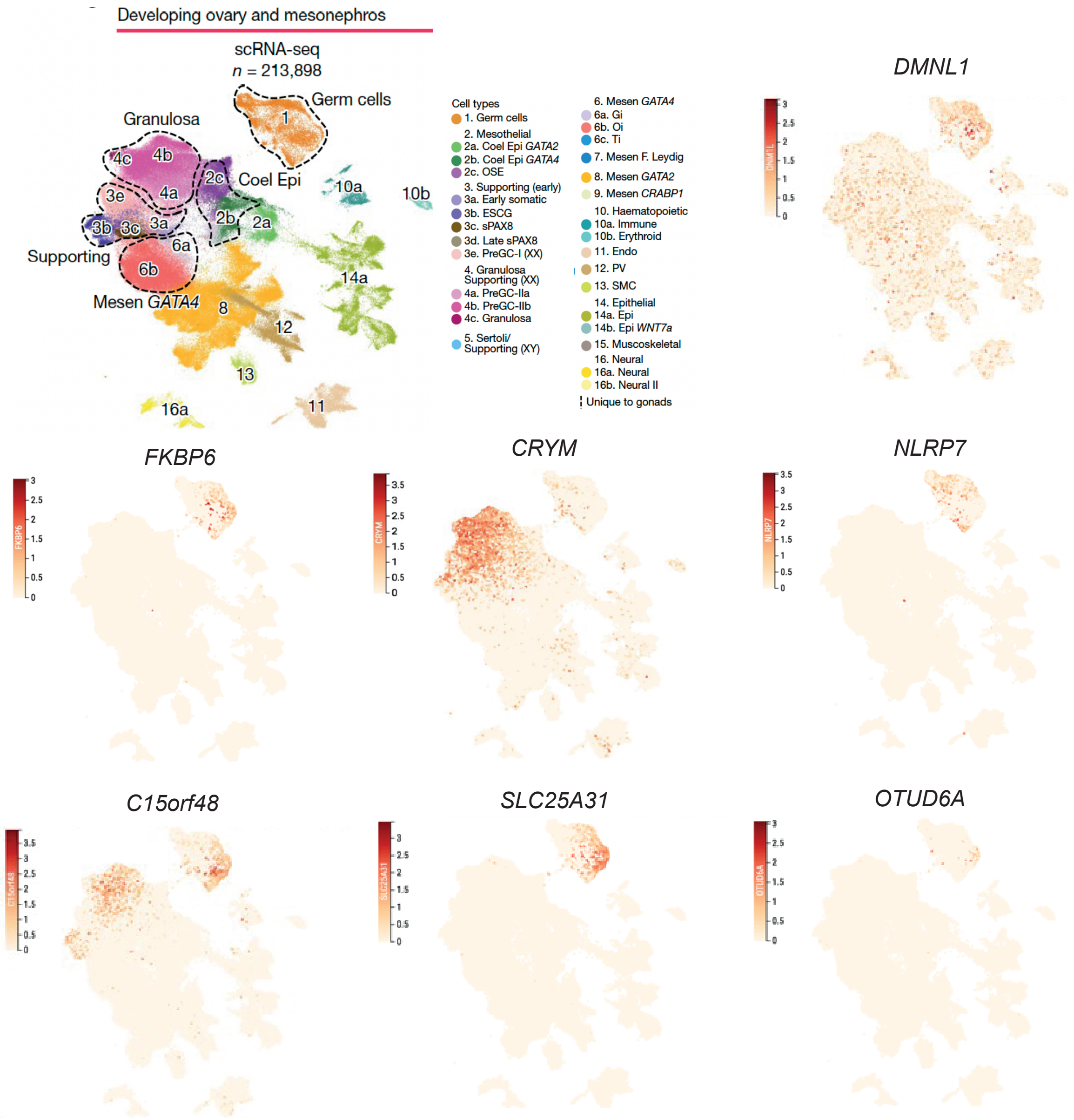

UMAP (uniform manifold approximation and projection) representation of human fetal ovary single-cell data from Garcia-Alonso et al (Garcia-Alonso et al, *Nature,* 2022; published CC-BY-4.0 licence) using the online interactive user tool at https://www.reproductivecellatlas.org (Roser Vento-Tormo, Wellcome Sanger Institute, Hinxton, UK). Key novel ovary-specific genes identified in this work are localised to the Garcia-Alonso et al data for validation.

Supplementary Figure 7: Validation of novel ovary-specific transcription factors using the Garcia-Alonso *et al* single-cell gonadal atlas

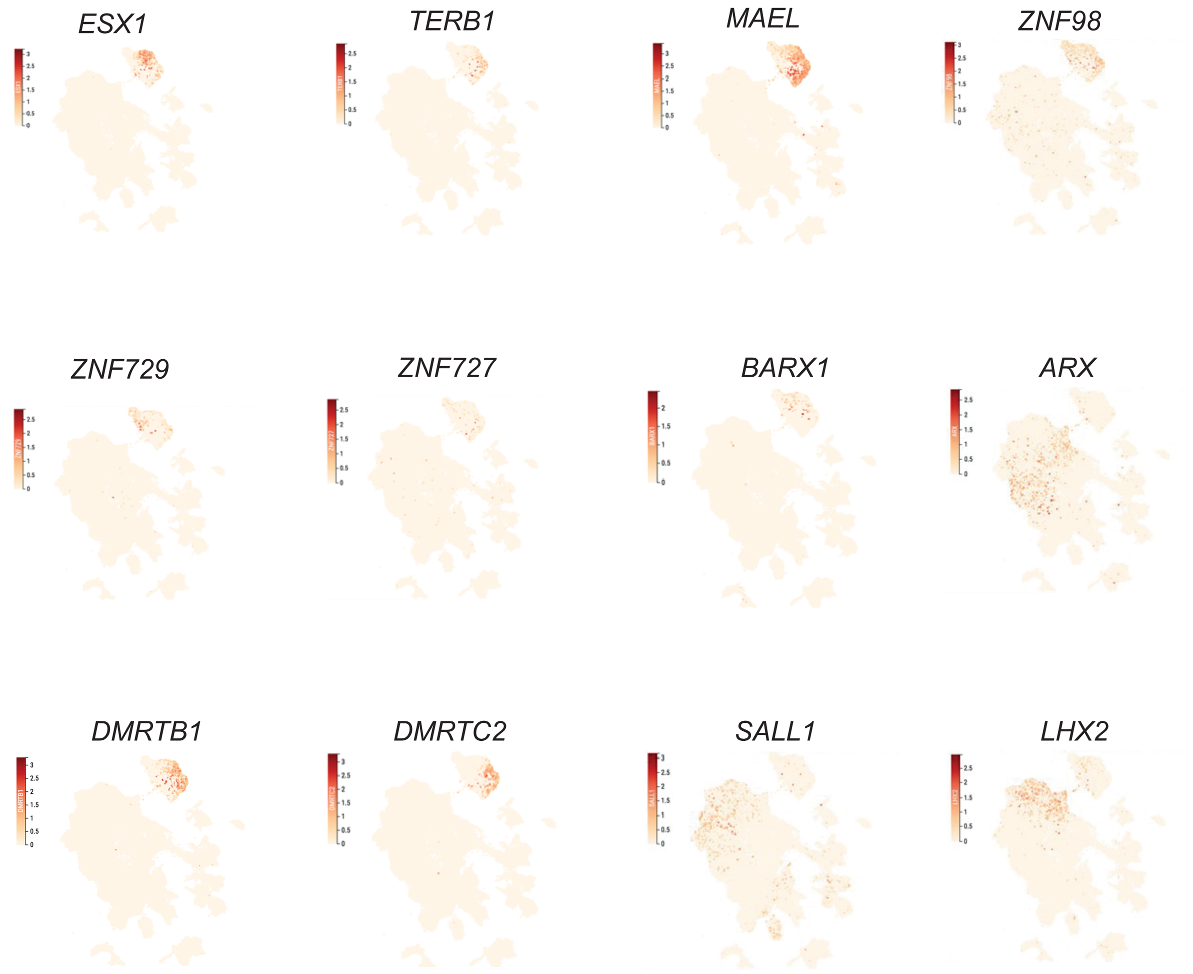

UMAP representation of human fetal ovary single-cell data from Garcia-Alonso et al (Garcia-Alonso et al, *Nature,* 2022) using the online interactive user tool at https://www.reproductivecellatlas.org (Roser Vento-Tormo, Wellcome Sanger Institute, Hinxton, UK). Key novel ovary-specific transcription factors identified in this work are localised to the Garcia-Alonso et al data for validation.

Supplementary Figure 8: Validation of selected novel meiosis factors using the Garcia-Alonso *et al* single-cell gonadal atlas

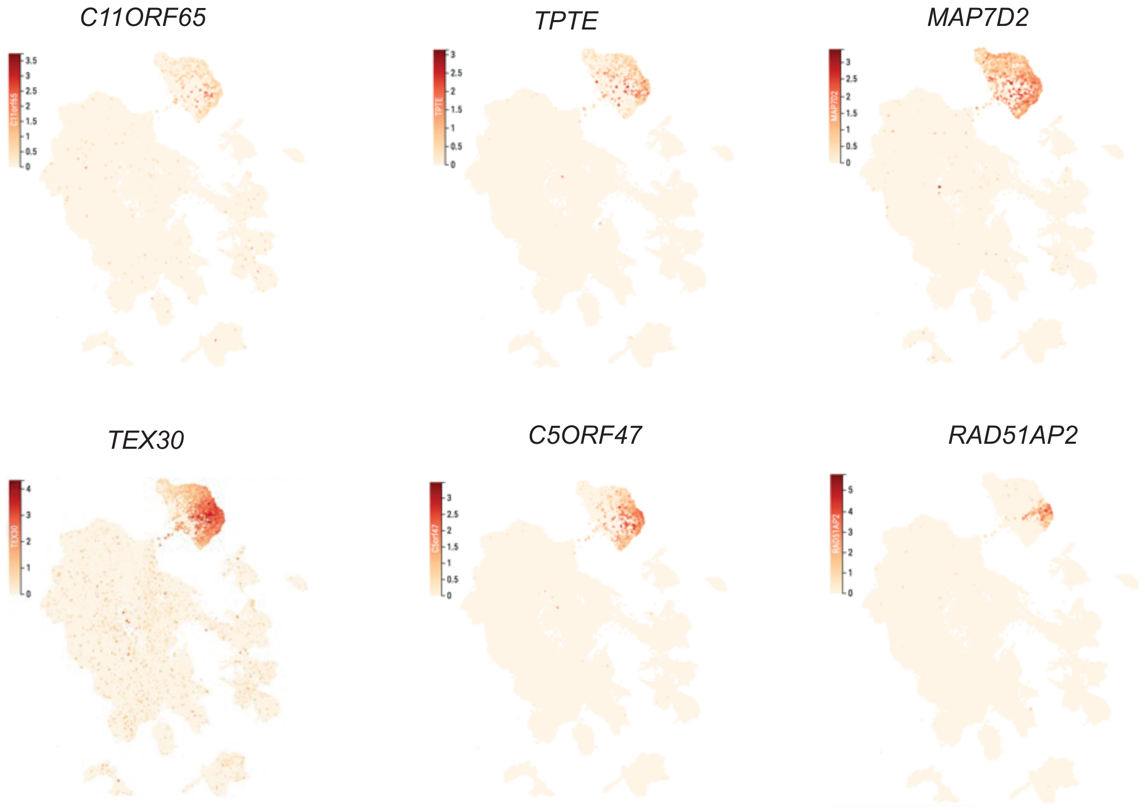

UMAP representation of human fetal ovary single-cell data from Garcia-Alonso et al (Garcia-Alonso et al, Nature, 2022) using the online interactive user tool at https://www.reproductivecellatlas.org (Roser Vento-Tormo, Wellcome Sanger Institute, Hinxton, UK). Key novel ovary-specific meiosis factors identified in this work are localised to the Garcia-Alonso et al data for validation.
